# Epigenetic Regulation of Erythropoiesis: From Developmental Programs to Therapeutic Targets

**DOI:** 10.3390/ijms26136342

**Published:** 2025-06-30

**Authors:** Ninos Ioannis Vasiloudis, Kiriaki Paschoudi, Christina Beta, Grigorios Georgolopoulos, Nikoletta Psatha

**Affiliations:** 1 Department of Genetics, Development and Molecular Biology, School of Biology, Aristotle University of Thessaloniki, 54124 Thessaloniki, Greecegeorgolog@gmail.com (G.G.); 2 Gene and Cell Therapy Center, Hematology Clinic-Bone Marrow Transplantation Unit, “George Papanikolaou” Hospital, 57010 Thessaloniki, Greece

**Keywords:** erythropoiesis, chromatin remodeling, transcription factors, hemoglobin switching, regulatory elements, epigenetic therapy, enhancer regulation

## Abstract

Erythropoiesis, the process driving the differentiation of hematopoietic stem and progenitor cells to mature erythrocytes, unfolds through tightly orchestrated developmental stages, each defined by profound epigenetic remodeling. From the initial commitment of hematopoietic progenitors to the terminal enucleation of erythrocytes, dynamic changes in chromatin accessibility, transcription factor occupancy, and three-dimensional genome architecture govern lineage specification and stage-specific gene expression. Advances in our understanding of the regulatory genome have uncovered how non-coding elements, including enhancers, silencers, and insulators, shape the transcriptional landscape of erythroid cells. These elements work in concert with lineage-determining transcription factors to establish and maintain erythroid identity. Disruption of these epigenetic programs—whether by inherited mutations, somatic alterations, or environmental stress—can lead to a wide range of hematologic disorders. Importantly, this growing knowledge base has opened new therapeutic avenues, enabling the development of precision tools that target regulatory circuits to correct gene expression. These include epigenetic drugs, enhancer-targeted genome editing, and lineage-restricted gene therapies that leverage endogenous regulatory logic. As our understanding of erythroid epigenomics deepens, so too does our ability to design rational, cell-type-specific interventions for red blood cell disorders.

## 1. Introduction

Erythropoiesis is the vital process responsible for the production of red blood cells (RBCs), ensuring the continuous supply of cells capable of transporting oxygen from the lungs to tissues throughout the body. To meet the changing oxygen demands and maintain adequate red cell numbers, erythropoiesis undergoes dynamic regulation during development, progressing through distinct embryonic, fetal, and adult stages. During the embryonic stage, primitive erythropoiesis originates in the yolk sac, producing large, nucleated RBCs that circulate briefly within the early embryo. These primitive cells mature within the vasculature and ultimately undergo enucleation to support embryonic growth. Conversely, definitive erythropoiesis, located in the fetal liver and later in the bone marrow, generates smaller, enucleated RBCs that maintain oxygen delivery throughout fetal development and after birth [1,2,3,4,5]. 

Both primitive and definitive erythroid lineages arise from committed progenitor cells that progress through distinct precursor stages, culminating in enucleated mature RBCs [6]. Alongside the developmental transitions in erythropoiesis, profound changes occur at each stage of erythroid differentiation, characterized by dramatic cellular and molecular shifts. These transitions include global alterations in chromatin structure, dynamic remodeling of the transcriptional landscape, and the progressive activation and silencing of specific gene networks, all of which are orchestrated by a tightly controlled and stage-specific epigenetic regulatory network [6,7,8]. Owing to the extensive and highly coordinated changes in gene expression and chromatin dynamics, erythropoiesis provides an exceptional model system for studying the epigenome and the gene regulatory networks (GRNs) that govern lineage commitment and differentiation. Insights gained from these studies have not only deepened our understanding of normal erythroid biology but have also revealed critical mechanisms underlying various erythroid disorders such as β-thalassemia, sickle cell disease, Diamond–Blackfan anemia, congenital dyserythropoietic anemias, and myelodysplastic syndromes with erythroid predominance. These findings offer novel diagnostic markers and therapeutic targets to combat these diseases. In this review, we sought to highlight key aspects of the epigenetic regulation of erythropoiesis, discuss how these mechanisms contribute to erythroid health and disease, and explore their potential therapeutic implications.

## 2. Dynamic Chromatin Remodeling During Development and Lineage Commitment

During development and lineage commitment, cells must undergo profound changes in gene expression to acquire and maintain their specific identities. A central mechanism driving these transitions is extensive chromatin remodeling [9] and dynamic changes in chromatin interactions [10], which regulate the accessibility of genomic regions to transcriptional machinery and orchestrate precise gene activation and repression programs. In the context of erythropoiesis, chromatin remodeling is essential for guiding multipotent hematopoietic stem and progenitor cells (HSPCs) towards a committed erythroid fate. We and others have previously shown that this process involves coordinated changes in chromatin structure coupled with transcription factor binding that progressively restricts cellular potential and reinforces erythroid-specific gene expression programs [8,11]. 

### 2.1. The β-Globin Locus: A Model of Developmental Gene Regulation

**Figure 1:**
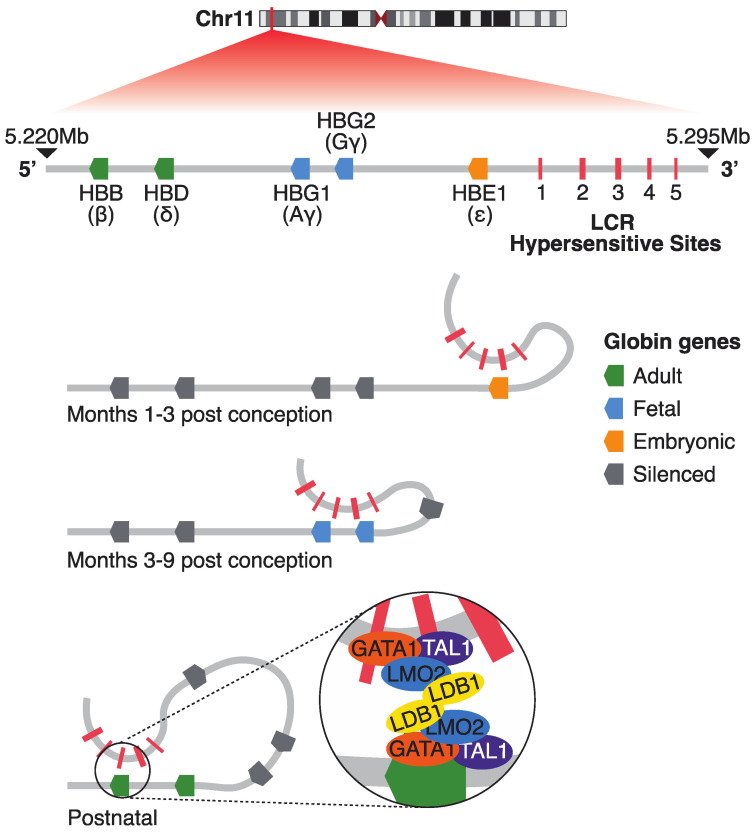
The human beta-like globin gene cluster along with the 5 hypersensitive sites of the locus control region (LCR) super-enhancer. The developmental control of the genes is mediated by the LCR, which interacts with different genes during embryonic, fetal, and postnatal/adult life. Finally, a scheme of the transcription factor complex which facilitates the interaction between the LCR and the adult globins is shown. Coordinates shown are in GRCh38 genome assembly.

Among the many genomic regions undergoing developmental dynamic epigenetic remodeling in erythropoiesis, the β-globin locus remains the most thoroughly characterized, offering a paradigm for understanding how chromatin architecture and enhancer/promoter communication regulate gene expression throughout development. During mammalian development, shifting oxygen demands trigger two hemoglobin switches, governed in part by the human β-globin locus through the stage-specific activation of ε, Gγ, Aγ, δ, and/or β genes located on chromosome 11 [12,13]. The expression of globin genes is tightly regulated during the developmental stages through the locus control region (LCR). Beta-globin LCR is located 6 to 20 kb upstream of the beta-globin genes and consists of five DNase I hypersensitive sites (Figure 1).

Each of these regions has a core sequence of 250 nucleotides filled with transcription factor motifs [14]. It is known that the HS1-HS4 have cell-type-specific enhancer activity that increases the expression of beta-like genes, whereas the HS5 acts as an insulator and has a CTCF (CCCTC-binding factor) binding site [13,15]. In the embryonic stage, the ε-globin gene (*HBE1*) is predominantly expressed, facilitated by an open chromatin structure at the LCR, characterized both by DNase I hypersensitivity [15,16] and active histone modifications such as H3K9 acetylation and H3K4 methylation [17]. At this stage, the LCR engages in chromatin looping interactions with the ε-globin promoter, a mechanism mediated by transcription factors including *GATA1* and *NF-E2* [18,19,20,21,22,23,24,25,26,27] (Figure 1).

As development proceeds to the fetal stage, transcriptional activity shifts to the γ-globin genes (*HBG1* and *HBG2*), while the LCR maintains an accessible configuration but reorients its enhancer/promoter interactions toward the γ-globin promoters [20]. This transition is marked by enriched H3K4me3 and histone acetylation at γ-globin regulatory regions, although repressive factors such as BCL11A begin to emerge later in fetal erythropoiesis, preparing for the eventual silencing of fetal globin expression [28,29,30].

In the adult stage, expression is predominantly restricted to the β-globin gene *(HBB)* (Figure 1), accompanied by repressive epigenetic modifications—such as H3K9me3 and DNA methylation—at embryonic and fetal gene promoters, and persistent activation marks at the β-globin promoter [28,30]. Notably, whether DNA methylation at embryonic and fetal globin promoters is a primary driver of gene silencing or a secondary consequence of transcriptional repression remains under active investigation; evidence supports both models, suggesting that promoter methylation may both reinforce and stabilize the hemoglobin switch once initiated by transcriptional regulators [31,32,33]. 

### 2.2. Chromatin Architecture and the Role of CTCF

In addition to transcriptional and epigenetic repression, higher-order chromatin organization, shaped by architectural proteins like CTCF, is crucial for the developmental regulation of the β-globin locus. CTCF is a key regulator of transcription, chromatin architecture, and genome organization. Notably, CTCF binding can be modulated by DNA methylation, linking changes in chromatin architecture to epigenetic state. Immortalized cells show widespread loss of CTCF binding linked to increased methylation despite upregulated CTCF expression, suggesting a compensatory mechanism that preserves global occupancy levels [34]. This methylation-dependent regulation of CTCF is particularly relevant to developmental gene control, such as in the hemoglobin switch. In this context, promoter methylation at embryonic and fetal globin genes may both initiate and reinforce transcriptional silencing. Furthermore, CTCF and cohesin complexes mediate higher-order chromatin architecture, promoting the formation of insulated domains that facilitate exclusive LCR-β-globin interactions [35,36,37]. 

### 2.3. Regulatory Mechanisms at the α-Globin Locus

**Figure 2:**
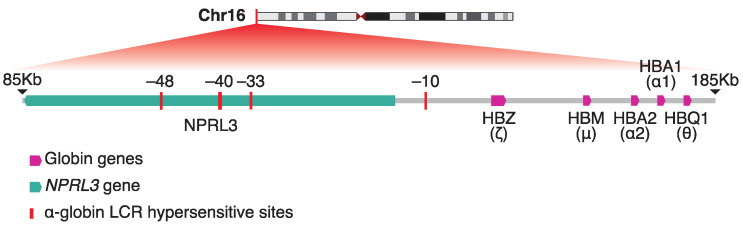
The human alpha-like globin gene cluster along with the identified hypersensitive sites of the locus control region (LCR) super-enhancer harbored in the neighboring *NPRL3* gene. Coordinates shown are in GRCh38 genome assembly.

Similar to the β-globins, the chromatin dynamics of the α-globin locus have been extensively studied. The alpha-globin locus is located at chromosome 16 in a G-C, Alu-dense, gene-dense segment of DNA. The alpha-globin locus encodes the alpha- and ζ-globin genes. The alpha-globin promoters have a NF-Y binding site (CAAT box) and binding sites for the transcription factors *GATA1* and *KLF1* [38,39]. Moreover, the promoters of all genes in alpha-globin locus contain GC-islands. The alpha-like genes are distal regulated by four cis-regulatory enhancer elements located 10 to 48kb upstream of the genes, known as alpha-globin LCR. There are four distinct DNaseI hypersensitive sites in the a-globin LCR, named MCS-R4 (multispecies conserved sequence R4), MCS-R3, MCS-R2, and MCS-R1 [40] (Figure 2). Among them the MCS-R2, known also as HS-40, is the most critical for alpha-globin expression. 

During the erythroid differentiation, the alpha-globin expression is tightly regulated through strong interactions between the alpha-globin LCR and the promoter of the corresponding gene through looping formation [41]. The transcription factors *GATA1*, nuclear factor-erythroid 2 (*NF-E2*), stem cell leukemia (SCL) pentameric complex, and *KLF1* play crucial roles in alpha-globin expression [39]. Notably, the alpha-globin expression is precisely regulated by specific epigenetics mechanisms. In non-erythroid cells, the polycomb repressive complex 2 (PRC2) binds to the alpha-globin promoter and induced H3K27me3 marks, promoting transcriptional silencing [42]. Conversely, during erythroid differentiation, the PRC2 is detached and the methylation marks are removed by demethylation enzymes, such as Jumonji domain-containing 3 and histone lysine demethylase (JMJD3) enzyme [43]. Capture-C experiments have shown that the α-globin locus also undergoes dramatic spatial re-arrangement during mouse erythroid differentiation [44]. Further studies have provided structural models of the different states of the region. The inactive α-globin locus in embryonic stem cells (ESCs) is characterized by a compact globular structure dominated by non-specific DNA-DNA contacts. In contrast, in the erythroid cells the region is re-arranged in a highly structured folder hairpin conformation which facilitates the contact between the α-globin genes and their enhancers in cis (i.e., on the same chromosome) [45], where increased frequency of enhancer/promoter interactions correlates with upregulation of α-globin expression [46].

### 2.4. Sequence Orientation and Cohesin Flow in Gene Regulation

Enhancers activate transcription when brought into spatial proximity with their target promoters. Unlike individual enhancers, which typically function in an orientation-independent manner, super-enhancers (SEs) and LCRs can exhibit orientation-dependent activity. In the case of the α-globin locus, active enhancers coincide with peaks of the cohesin complex and its loader Nipbl, indicating these regions may serve as cohesin entry sites in erythroid cells [47,48,49]. Cohesin translocation along chromatin can be impeded by elements like replication machinery or CTCF-bound insulators. The orientation of CTCF binding is critical; when the N-terminus of CTCF faces the enhancer, cohesin movement, and consequently enhancer function are preferentially blocked, leading to reduced gene expression [50]. Experimental inversion of CTCF sites and SEs within the α-globin cluster has demonstrated that the relative orientation of both elements influences transcriptional output [51]. Similar observations have been made at the β-globin locus, where inversion of the β-globin locus control region (LCR) significantly reduces β-globin expression [52]. Furthermore, the β-globin LCR, when inserted in different orientations among housekeeping genes, activates different sets of genes depending on its direction despite exerting bidirectional effects [53]. Recent findings demonstrated that forced chromatin looping of the β-globin locus control region (LCR) to the *HBG* promoters has shown to reactivate their expression in adult erythroid cells, demonstrating that physical juxtaposition of the LCR is sufficient to bypass promoter silencing. Deletions or inversions of non-insulating genomic regions can reduce linear distance between enhancers and promoters, effectively hijacking enhancer activity and enabling inappropriate gene activation [54]. Together, these findings highlight that while single enhancers function independently of orientation, SEs and LCRs may encode directional information that modulates their regulatory potential. Thus, the developmentally regulated chromatin dynamics at globin loci illustrate a sophisticated interplay between enhancer accessibility, chromatin looping, and stage-specific transcription factor recruitment.

### 2.5. Global DNA Methylation Dynamics in Erythropoiesis

Beyond chromatin accessibility and quaternary DNA structure dynamics, epigenetic regulation is facilitated through enzymatic DNA methylation, where changes in methylation patterns can activate or repress transcription. Genome-wide DNA methylation studies in both human and mouse erythropoiesis uncover global dynamic methylation patterns that govern erythroid development and differentiation [7,55,56]. Specifically, transition from HSPCs to mature erythroid precursors is characterized by gradual yet continuous global DNA demethylation involving dynamic interplay between DNA methyltrasferase *DNMT1* and the de novo methylases *DNMT3A* and *DNMT3B* [55,56]. Interestingly, dramatic changes in methylation profiles occurred in promoters of genes that were not expressed throughout the erythropoiesis or newly silent genes which were mostly associated with myeloid and lymphoid fates, suggesting that methylation programs establish erythroid fate through lineage restriction [56]. In parallel, developmental comparisons of human erythroblasts derived from fetal liver versus adult bone marrow revealed over 5900 differentially methylated CpGs, many of which cluster within erythroid enhancers and transcription factor binding motifs [57]. Surprisingly, the majority of differentially methylated regions were hypermethylated in fetal erythroblasts, counter to the expectation of a globally more open fetal epigenome. These CpGs were strongly enriched in proximity to erythroid-specific enhancers and near genes with known or newly suggested roles in red blood cell differentiation and proliferation. Notably, many of these regions were associated with transcription factor binding motifs relevant to erythropoiesis, underscoring a developmentally staged rewiring of the epigenome that likely fine-tunes enhancer activity and gene expression during erythroid maturation.

## 3. Role of Erythroid Transcription Factors in Shaping Accessible Chromatin Landscapes

The temporal interplay between chromatin and transcription factors (TFs) plays a central role in shaping transcriptional programs during development and differentiation. In adult human erythropoiesis, this dynamic relationship has been comprehensively characterized using multiomic approaches, which profile chromatin accessibility and gene expression across erythroid lineage progression [8,11]. These studies revealed that erythroid differentiation is orchestrated by a series of discrete, temporally resolved regulatory modules composed of transcription factors and their target chromatin elements. These modules exhibit stage-specific activity and reflect the transition from hematopoietic stem and progenitor cells (HSPCs) through lineage restriction to terminal erythroid maturation. By integrating dense temporal chromatin accessibility and gene expression profiling, we recently identified a limited set of approximately 50 transcription factors that sequentially engage the regulatory landscape, underscoring a surprisingly compact regulatory architecture [8]. Single-cell proteomics along the ex vivo differentiation of red blood cells from HSPCs highlights gradients in protein abundance of co-expressed key transcription factors as the underlying mechanism of erythroid lineage specification. Specifically, gradual quantitative changes in the relative abundance of *KLF1* and *FLI1* can guide hematopoietic progenitors towards the erythroid or the megakaryocytic lineage, respectively [58].

The effects of regulatory variants on erythropoiesis ultimately converge on the gene regulatory networks controlled by erythroid transcription factors. These master regulators, such as *GATA1*, *TAL1*, and *KLF1*, not only activate lineage-specific genes but also orchestrate chromatin accessibility and three-dimensional genome organization. Understanding their roles is essential to decipher how the erythroid epigenome is established, maintained, and perturbed in health and disease.

### 3.1. The GATA2-to-GATA1 Switch: Coordinating Erythroid Lineage Progression

**Figure 3:**
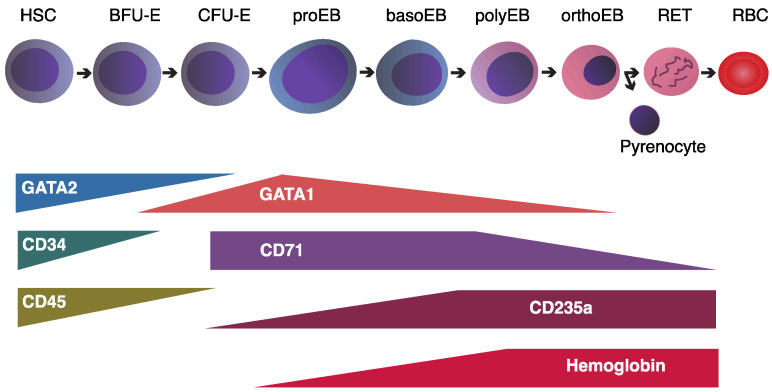
Schematic representation of human erythroid differentiation and associated markers. Progression from hematopoietic stem cells (HSCs) through erythroid progenitor stages, including burst-forming unit–erythroid (BFU-E), colony-forming unit–erythroid (CFU-E), proerythroblasts (proEB), basophilic erythroblasts (basoEB), polychromatic erythroblasts (polyEB), and orthochromatic erythroblasts (orthoEB), culminating in reticulocytes (RET), pyrenocytes, and mature red blood cells (RBCs). Key transcription factors (*GATA1*, *GATA2*), surface markers (CD34, CD45, CD71, CD235a), and hemoglobin expression are indicated at relevant stages.

*GATA1* and *GATA2* transcription factors play key roles in gene regulation during erythropoiesis (Figure 3).

They are both zinc-finger DNA-binding proteins and upon their expression *GATA1* and *GATA2* bind to (A/T)GATA(A/G) motifs. *GATA1* and *GATA2* are strictly regulated in a cell-specific manner [59]. *GATA2* supports HSCs proliferation and prevents premature differentiation. During the embryonic development *GATA2* regulates embryonic blood formation in the yolk sac and liver [60,61]. *GATA1* is more active at the later stages of differentiation. *GATA1* regulates globin genes expression and transactivates the erythropoietin receptor gene [62,63], enzymes for heme synthesis, and proteins that make up the erythroid cell membrane. Also, is necessary for megakaryocyte maturation and platelet formation [64,65], as loss of *GATA1* disrupts the proper maturation of erythroid and megakaryocyte cells, highlighting its essential role in ensuring correct blood cell lineage development [66,67,68]. Moreover, *GATA1* interacts with several cofactors, including *FOG1*, PU.1, and the transcriptional coactivators p300/CBP, which enhance its regulatory function [67,69,70]. *FOG1* (Friend of *GATA1*) is an essential cofactor that binds directly to *GATA1*, enabling it to activate or repress target genes required for erythroid and megakaryocyte (platelet precursor) development. The *GATA1*/*FOG1* interaction is critical for normal blood cell maturation; disruption of this interaction impairs erythroid and megakaryocyte development and is linked to blood disorders [71,72,73,74,75]. *GATA1* promotes erythroid differentiation, while PU.1 drives myeloid lineage development. These factors can physically interact and inhibit each other’s function, creating a genetic switch that determines cell fate [71,76,77]. *FOG1* also represses PU.1 expression by recruiting *GATA1* to the PU.1 promoter, thereby promoting erythroid over myeloid differentiation [78]. *GATA1* undergoes acetylation by p300/CBP on several clusters of lysine residues adjacent to the two DNA-binding zinc fingers [79,80]. Mutating these sites dramatically impairs *GATA1* function, suggesting that acetylation of *GATA1* plays a role in its transcriptional activity. Notably, *GATA1* plays crucial role to *GATA2* downregulation. Genome-wide binding analysis in mouse fetal liver cells revealed that *GATA1* strongly binds the *GATA2* locus in both early and late erythroid stages and that this binding is associated with increased repressive histone marks, including H3K27me3, specifically in downregulated target gene clusters [81]. *GATA2* promotes its own expression by binding to the upstream WGATAR motif. During erythroid differentiation, the increased production of *GATA1* recruits the *FOG1* and the NuRD complex, leading to the formation of the *GATA1*-*FOG1*-NuRD complex. This complex effectively occupies on WGATAR motif, resulting in *GATA2* downregulation [82,83,84,85].

### 3.2. TAL1: Early Hematopoiesis and β-Globin Regulation

*TAL1* (also known as SCL) is a basic helix–loop–helix transcription factor that plays a vital role in the early stages of blood cell formation (hematopoiesis). In mice lacking *TAL1*, embryonic development is halted due to a complete failure of hematopoiesis, and embryonic stem cells without *TAL1* are unable to contribute to any blood cell lineages in adult chimeric models [86,87]. At the molecular level, *TAL1* exerts its function by forming dimers with E proteins such as E12 and E47, enabling it to bind to E-box motifs in the regulatory regions (promoters and enhancers) of target genes, thereby influencing their transcription [88,89]. This mechanism is critical for regulating genes that control blood cell proliferation, survival, and differentiation. However, abnormal *TAL1* expression, often due to chromosomal translocations or other genetic alterations, has been linked to blood cancers, particularly T-cell acute lymphoblastic leukemia (T-ALL) [90,91]. Understanding both *TAL1*’s role in normal blood development and its contribution to leukemia highlights its significance as a potential target for therapeutic intervention in hematologic disorders. *TAL1* also collaborates with several cofactors—such as *LMO2*, *GATA1*, *LDB1*, and *RUNX1*—to form transcriptional complexes that enhance its regulatory precision and biological effectiveness in hematopoiesis [87,92,93]. In the context of hemoglobin regulation, *TAL1* and *GATA1*, *LMO2* complex forces long-range interaction between β-globin LCR and active globin genes. *TAL1* knockdown in K562 cells decreased the binding of *LDB1* and *LMO2* in the LCR locus and resulted in a consequent gamma-globin depression. In contrast, overexpression of *TAL1* increased the interaction between β-globin LCR and the ^G^γ-globin gene and increased gamma-globin expression [94].

### 3.3. KLF1: Terminal Erythroid Differentiation and Hemoglobin Switching

*KLF1*, also known as erythroid Krüppel-like factor (*EKLF*), is a transcription factor that binds specifically to the DNA sequence CCM-CRC-CCN via its C2H2-type zinc finger domains [95]. *KLF1* is involved in several steps of hematopoiesis, including cell-cycle regulation, the central macrophages of erythroblastic island function, and the erythroid commitment [96,97,98,99,100]. It is found to be most essential for the final stages of erythroid cell development by regulating essential erythroid genes involved in erythroid cell proliferation, heme biosynthesis, and membrane integrity [100,101,102,103,104,105]. *KLF1* has also been identified as a key player in globin regulation through simultaneously binding on the β-globin LCR and on the CACCC box at the β-globin promoter [106,107,108,109]. Moreover, *KLF1* interacts with CBP/p300 and BRG1 components of SWI/SNF complex [110]. The loop formation between the β-globin LCR and the β-globin promoter induces β-globin production. Disruption of the *KLF1* gene in mice impairs hemoglobin synthesis and causes a severe form of β-thalassemia, highlighting its critical function in erythropoiesis [111]. Studies using transcriptomic profiling of *KLF1*-deficient mice have revealed major alterations in gene networks responsible for controlling the cell cycle [100]. Specifically, the absence of *KLF1* leads to defective progression into the S phase during red blood cell formation, reinforcing its essential role in coordinating proper cell cycle dynamics during erythropoiesis [112].

## 4. Non-Coding Variation and Erythroid Phenotypes

The dynamic chromatin remodeling that underlies erythropoiesis highlights the critical role of regulatory DNA elements in controlling gene expression. Genetic variation in these regulatory regions, particularly single-nucleotide polymorphisms (SNPs), can subtly alter transcription factor binding, chromatin accessibility or enhancer/promoter communication, thereby modulating erythroid gene expression programs [113]. 

### 4.1. Functional Consequences of Distal Regulatory Variants

Variants in distal regulatory elements (REs) have been less explored than those in promoters or 3′ UTRs, largely because their genomic locations are less predictable. The functional impact of mutations in the aforementioned regions depends on specific genomic contexts, including binding site alterations and enhancer sensitivity to sequence variation [114,115]. This indirect and cumulative influence makes it especially difficult to pinpoint which genes are affected, how regulation is altered, and what the biological consequences are, particularly when compared to coding variants [116]. CRISPR/Cas9-mediated loss-of-function studies offer a direct way to test the functional impact of non-coding mutations and regulatory SNPs associated with monogenic erythroid disorders. Combined with experimental and bioinformatic analyses, this approach has been used to generate mutational maps that predict the effects of variants across CREs. It has also helped reveal the functional hierarchy of constituent enhancers and provide critical insights into their complexity [117,118].

### 4.2. Therapeutic Relevance of Regulatory SNPs

Deciphering the functional consequences of regulatory SNPs is critical for advancing therapies that target gene regulatory networks. When regulatory SNPs disrupt gene expression or pathway activity, they can reveal potential therapeutic targets, enabling the development of treatments that can restore normal protein levels and ameliorate disease. A clear example of this is seen in the treatment of sickle cell disease and β-thalassemia, where genome-editing therapeutic strategies have emerged based on our understanding of fetal hemoglobin (HbF) regulation. Hereditary persistence of fetal hemoglobin (HPFH), a term introduced in 1958, refers to naturally occurring deletions or point mutations in the β-globin gene cluster that allow continued expression of HbF into adulthood [119]. The resulting distribution of HbF across red blood cells varies. When HbF is present only in a subset of cells, the condition is termed heterocellular HPFH; in contrast, pancellular HPFH refers to a more uniform expression across most or all red blood cells [120].

This difference in distribution likely reflects the strength of the γ-globin gene expression driven by a specific mutation [120]. A well-characterized non-coding SNP (ncSNP) example is the C > T polymorphism at rs7482144 in the *HBG2* promoter, which is associated with elevated HbF levels through disruption of transcriptional repression. This variant interferes with the binding of BCL11A and ZBTB7A, which typically occupy motifs located approximately 115 bp and 200 bp upstream of the transcription start site, respectively [121,122,123,124]. Disruption of the *HBG2* promoter at rs7482144 (158 bp upstream) increases HbF, though to a lesser extent than mutations directly affecting the ZBTB7A or BCL11A binding motifs, likely because it only impacts *HBG2* rather than both γ-globin genes [125,126]. The diversity in HbF levels observed across different point mutations may depend on how they alter transcription factor binding or protein–complex interactions. Notably, when rs7482144 co-occurs in cis (i.e., on the same locus and allele) with other mutations (e.g., 175 T > C or 202 C > G), HbF levels can rise dramatically, up to 41% in some HbS heterozygotes, suggesting synergistic effects from the combined disruption of repressor binding and potential recruitment of activators such as *GATA1* [120,127,128]. The HBG promoter is not the only region where mutations have an impact in gamma globin expression. Various quantitative trait loci (QTL) have been associated with persistence of elevated HbF, including BCL11A, HBS1L-MYB, and the *HBB* gene cluster in general [121]. Among these, ncSNPs implicated in elevated HbF levels include rs4671393 in the BCL11A locus, as well as rs9399137 and rs9494142 in the HBS1L-MYB region [121]. In addition to HbF levels, certain ncSNPs can also affect other hematological parameters, including RBC count and size, platelet count, and hemoglobin levels [129]. For instance, rs9399137—a well-known ncSNP in the HBS1L-MYB—is associated with fewer RBCs and monocytes but larger, hemoglobin-rich RBCs and higher platelet counts [130]. Overall, SNPs are crucial genetic markers for managing β-thalassemia and SCD. Primarily, they help predict clinical severity by their association with HbF levels, which directly informs patient management, such as the timing of blood transfusions or decisions about hematopoietic stem cell transplantation [131,132]. A key example of this is seen in the β-globin gene cluster haplotypes in SCD. The Arab-Indian and Senegal haplotypes, for instance, are associated with elevated HbF, leading to milder symptoms, whereas the Benin and Bantu haplotypes are linked to lower HbF and more severe disease [133].

In addition to predicting overall disease severity, certain SNPs also serve as markers for specific complications. For instance, variants in the *TLR1/TANK* and *MALT1* genes have been associated with an increased risk of alloimmunization in some populations [134]. Others, such as rs7319269, are linked to a higher risk of acute chest syndrome, although the underlying mechanisms are still being investigated [135,136].

Finally, mapping these genetic variations enhances diagnostic accuracy and supports informed reproductive choices through early and noninvasive prenatal screening [137,138].

### 4.3. Non-Coding Variants in Transcription Factor Binding Sites

Non-coding SNPs can also give rise to both inherited and acquired erythroid disorders through mutations in genes encoding transcription factors and epigenetic regulators [138]. Mutations in *GATA1*, a master regulator of erythropoiesis and megakaryopoiesis, illustrate how disruptions in transcription factors or their DNA binding sites can impair blood cell development. For instance, missense mutations in *GATA1* cause anemia and thrombocytopenia, highlighting its essential role in hematopoiesis. The SNP rs311103, located in the XG gene region (Xg blood group), disrupts a *GATA1*-binding motif upstream of the gene. This disruption abolishes the expression of the Xga blood group antigen on erythrocytes, resulting in the Xg(a−) phenotype [139]. Two SNPs within the *GATA2*, rs2335052 and rs78245253, are associated with an increased risk of acute myeloid leukemia (AML) in Chinese populations [140]. Pathogenic variants in *GATA1*-binding motifs have also been implicated in several inherited erythroid disorders, including congenital dyserythropoietic anemia type II, X-linked sideroblastic anemia, and pyruvate kinase deficiency [118,141,142]. In vitro, CRISPR-mediated disruption of non-coding variants associated with X-linked sideroblastic anemia and pyruvate kinase deficiency revealed that even small (2–4 nt) changes in *GATA1* binding sites reduce *GATA1* binding and impair recruitment of cofactors like *TAL1* [118]. In contrast, mutations in *TAL1* binding sites have milder effects on *GATA1* binding but still alter gene expression, highlighting distinct roles for different transcription factors. In vitro, CRISPR-mediated disruption of non-coding variants associated with X-linked sideroblastic anemia and pyruvate kinase deficiency revealed that even small (2–4 nt) changes in *GATA1* binding sites reduce *GATA1* binding and impair recruitment of cofactors like *TAL1* [118]. In contrast, mutations in *TAL1* binding sites have milder effects on *GATA1* binding but still alter gene expression, highlighting distinct roles for different transcription factors [93]. Further bioinformatic analysis of multiple regulatory elements near genes in RBC structure, metabolism, and function supports a model where independent elements coordinate gene expression, mitigating the impact of single-site mutations [118].

Non-coding mutations have also been implicated in diagnostically challenging cases of Diamond–Blackfan anemia syndrome (DBAS), a disorder defined by pure red cell aplasia and early-onset macrocytic or normocytic anemia, typically caused by coding mutations in ribosomal protein genes. In cases where conventional testing, including targeted panels and exome sequencing, failed to identify a causative variant, non-coding regions have revealed pathogenic variants, particularly through in-depth reanalysis of whole-genome sequencing data [122]. Additionally, germline N-terminal truncating mutations in *GATA1*, producing the short isoform *GATA1*s, result in an inherited erythroid failure that phenotypically resembles DBAS [143,144]. 

Somatic mutations in the *GATA1* gene can lead to the production of a truncated protein called *GATA1*s that lacks the N-terminal domain. In children with Down syndrome (DS), the combination of trisomy 21 and *GATA1*s significantly increases the risk of developing transient abnormal myelopoiesis (TAM), a pre-leukemic disorder characterized by abnormal proliferation of immature megakaryoblasts due to blocked erythroid differentiation. While most TAM cases resolve spontaneously, about 10–30% progress to myeloid leukemia associated with Down syndrome (ML-DS) [145,146,147,148,149]. In the TAM cases that progress to ML-DS, additional somatic mutations occur in genes such as those encoding the cohesin complex (for example, STAG2), epigenetic regulators, or signaling molecules. These mutations enhance self-renewal and further block differentiation, ultimately cooperating with *GATA1*s to drive overt leukemia [148].

The miR-144/451 cluster, activated by the transcription factor *GATA1*, is essential for erythroid maturation and differentiation. miR-451 promotes red blood cell development by downregulating *GATA2*, a stem-cell-associated transcription factor, particularly in zebrafish [150,151]. Additionally, the cluster suppresses Myc to ensure proper erythroid differentiation [152]. Together, the *GATA1*–miR-144/451–*GATA2*/Myc regulatory axis plays a crucial role in erythropoiesis, and disruption of this pathway can result in impaired red blood cell formation and anemia.

### 4.4. Somatic Non-Coding Mutations in Hematologic Malignancies

Besides hereditary conditions, acquired mutations in erythroid REs can also contribute to hematologic malignancies [113]. Emerging evidence points to a critical role of non-coding regulatory variants as cancer drivers [153,154]. For instance, in the context of blood malignancies, somatic mutations can introduce de novo binding motifs for the MYB transcription factor at specific non-coding sites upstream of the *TAL1* proto-oncogene. MYB binds to these newly created sites and recruits its H3K27 acetyltransferase coactivator CBP, along with core components of a major leukemogenic transcriptional complex, including *RUNX1*, *GATA3*, and *TAL1* itself. This assembly drives the formation of an oncogenic super-enhancer that aberrantly activates *TAL1* expression, promoting leukemogenesis [124]. One of the most typical myeloproliferative neoplasms (MPNs) is Polycythemia Vera (PV). PV is characterized by excessive proliferation across all three hematopoietic lineages as well as extramedullary hematopoiesis, leading to elevated red blood cells and hematocrit [155]. The onset of the PV is primary driven by somatic mutations in JAK2 coding regions [156]. Additionally, mutations in non-coding regulatory regions have been associated with the development and/or the severity of PV. Namely, mutations in the enhancer’s region near to the JAK2 locus may influence chromatin loop formation, altering JAK2 expression [157]. Common non-coding germline SNPs in the TERT locus have been associated with MPN predisposition. Among all, the rs2736100_C is the most implicated with PV and is associated with longer telomeres and high blood numbers [158]. Moreover, variants in non-coding regions of SH2B3, *GATA2* and MECOM may alter hematopoietic stem/progenitor cell behavior and JAK2-mutant clone expansion. In addition to the well-known driver mutations, myeloproliferative neoplasms (MPNs) frequently harbor mutations in genes associated with epigenetic regulation and mRNA splicing. Mutations in *TET2*, *ASXL1*, *DNMT3A*, and *EZH2* are commonly found in patients with myeloproliferative neoplasms (MPNs). Loss-of-function mutations in the aforementioned genes led to global epigenetic dysregulation leading to an apparent induction of oncogenes and HSC self-renewal genes [154,159,160,161].

## 5. Leveraging Gene Regulation for Translational Applications

Understanding how erythroid transcription factors and chromatin landscapes regulate gene expression has not only clarified fundamental principles of erythropoiesis but also revealed a rich repertoire of regulatory elements that can be both targeted and harnessed for therapeutic purposes. Building on these insights, translational research is increasingly focused on leveraging key enhancers, silencers, and elements of chromatin architecture, not only to correct gene expression defects but also to reprogram erythroid gene networks in a disease-specific manner. In this section, we highlight how the therapeutic modulation and strategic use of the erythroid regulatory genome is driving innovation across a spectrum of hematologic diseases.

### 5.1. Erythroid-Specific Enhancers for Gene Therapy

Precise spatial and temporal control of gene expression is a cornerstone of effective gene therapy. Cis-regulatory elements can be used to fine-tune transgene expression, ensure lineage fidelity, and integrate seamlessly with endogenous regulatory networks.

Erythroid-specific expression of a β-globin transgene has been considered critically important for gene therapy of hemoglobinopathies since the earliest preclinical trials, when the importance of its regulatory machinery was already established [162,163,164,165,166,167]. The incorporation of critical components of the β-globin LCR into gene therapy vectors marked a pivotal advancement in the effort to achieve high-level, erythroid-specific expression of therapeutic transgenes [168,169,170,171], with the first successful design termed TNS9, achieving efficient gene transfer into murine HSPCs and sustaining high-level, erythroid-specific expression in irradiated mice receiving β-thalassemia marrow transplants [169]. Most current gene therapy for hemoglobinopathies relies on a truncated β-globin LCR spanning a ~3 kb in size to ensure both therapeutic and erythroid-specific expression of the respective transgene [172,173,174]. The first clinical trial for β-thalassemia [175] was performed using the Lentiglobin HPV569 vector, which contained a minimal β-globin promoter and the HS2, HS3, and HS4 elements. To avoid positional variegation and insertional genotoxicity, two copies of the chicken β-globin cHS4 insulator were initially inserted into the viral LTRs, but this introduction significantly reduced viral titers [176]. In the most recent version of this vector, Lentiglobin BB305 (created by Bluebird Bio-BBB), the cHS4 element was excluded, resulting in higher vector titers and improved gene transfer efficiency, enhancing its clinical utility [169]. Other insulating elements, of human origin, better efficiency and smaller size have been recently proposed [177,178]. The GLOBE LV, a beta-globin vector of similar design but containing a minimal HS2-HS3-LCR, demonstrated therapeutic efficacy in both murine models and patient-derived CD34+ cells, restoring HbA levels and reducing apoptosis during erythroid differentiation [173,179]. Recently, high-resolution mapping of the beta-globin LCR has allowed for the functional identification of enhancer elements suggesting alternative vector designs are feasible [180]. Mini LCR elements often contain binding sites for *GATA1*, *NF-E2*, and *EKLF*, which promote open chromatin and high-level, position-independent expression of the β-globin genes [181,182]. Some studies have shown that tandem copies of these short enhancer elements can significantly increase transgene expression by promoting an open chromatin structure at the promoter region [182]. However, optimal activity often requires cooperation between enhancer/LCR elements and promoter or intronic elements, highlighting the importance of context and sequence composition [183,184]. LCR free vectors employing alternative beta-globin locus enhancers have also been described. Building on previous work which identified such regions within the globin locus [185,186,187], vectors bearing the Aγ-globin gene have also been described [188,189].

Enhancers and promoters from other erythroid-expressed genes, such as ANK1 (Ankyrin-1), have also been characterized for their robust activity in erythroid cells and incorporated into vector designs [190,191]. Additionally, *KLF1*-bound enhancers have been explored, leveraging the erythroid-restricted transcription factor *KLF1* to drive lineage-specific activation of therapeutic genes [192,193,194]. A recent study developed a clinical-grade lentiviral gene therapy vector for Diamond–Blackfan anemia (DBA) that uses an engineered human *GATA1* enhancer (hG1E) to drive regulated expression of the transcription factor *GATA1* [195]. The hG1E enhancer was constructed by concatenating three chromatin regions that are selectively accessible during erythroid differentiation, enabling precise expression of *GATA1* only after lineage commitment and thus avoiding adverse effects on hematopoietic stem cells. This enhancer-based regulation successfully restored erythropoiesis in DBA models and patient samples, establishing a broadly applicable therapeutic strategy independent of the patient’s specific genetic mutation [195].

Recent research has focused on identifying compact, potent, and lineage-specific enhancer elements capable of recapitulating the activity of the β-globin locus control region (LCR) in a much smaller sequence. High-throughput screening of short (~200 bp) DNA fragments derived from developmentally active DNase I hypersensitive sites (DHSs) during human erythropoiesis has revealed several candidates that can effectively replace the μLCR in therapeutic vectors. Among these, two erythroid-specific enhancers were identified that exhibit minimal activity in non-erythroid lineages and closely mirror the temporal activation dynamics of native erythroid enhancers [196]. One of these enhancers is located within an intron of the *PVT1* locus. It is enriched for erythroid transcription factor binding motifs, including *GATA1*, *TAL1*, and *KLF1*, and becomes accessible early during erythroid differentiation, reflecting the activation pattern of its endogenous DHS. Deletion of the enhancer leads to a marked reduction in *PVT1* transcription, confirming its regulatory role, despite limited evidence implicating the PVT1 lncRNA in erythropoiesis. The second enhancer originates from an exonic DHS within a gene encoding the Peroxisome Proliferator Activated Receptor Alpha, *PPARA*. Like the PVT1-derived element, it is enriched for erythroid transcription factor motifs but exhibits peak activity at later stages of erythroid maturation [196]. *PPARA* itself has been identified as an erythroid-expressed gene, further supporting the relevance of this enhancer to red blood cell development [197,198]. 

Overall, erythroid-specific enhancers, from the canonical β-globin LCR to novel elements like *PPARA* or hG1E, have become essential tools in gene therapy, enabling precise, lineage-restricted transgene expression that enhances efficacy while minimizing off-target effects.

### 5.2. Fetal Hemoglobin Reactivation

**Figure 4:**
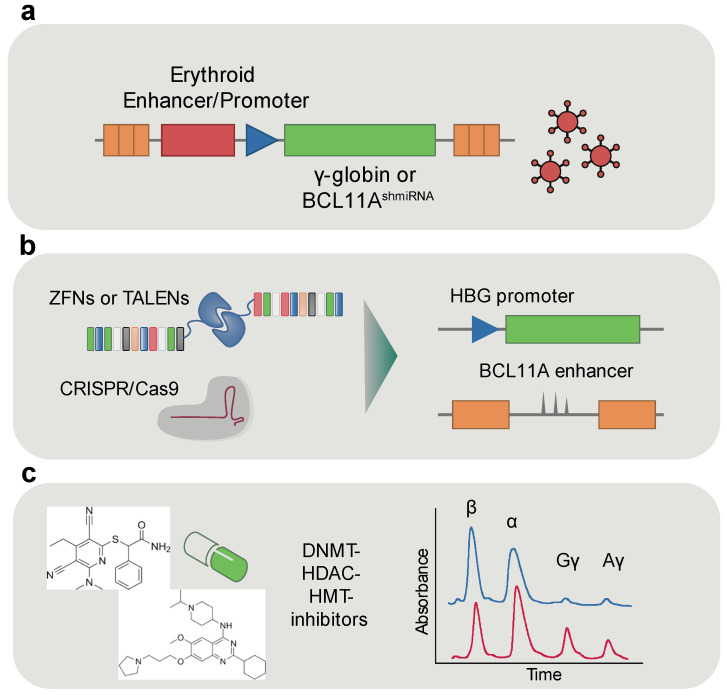
Therapeutic strategies for fetal hemoglobin (HbF) induction in β-hemoglobinopathies. Schematic overview of current approaches to reactivate γ-globin expression as a treatment for β-thalassemia and sickle cell disease: (**a**) Gene addition therapy using lentiviral vectors expressing γ-globin or short hairpin RNAs (shRNAs) targeting the HbF repressor BCL11A; (**b**) genome editing strategies that disrupt BCL11A expression by targeting its erythroid-specific enhancer or introduce mutations in the HBG promoters to mimic hereditary persistence of fetal hemoglobin (HPFH); (**c**) pharmacological induction through the use of epigenetic modulators such as DNA methyltransferase (DNMT) inhibitors, histone deacetylase (HDAC) inhibitors, and histone methyltransferase (HMT) inhibitors, which reactivate γ-globin expression via chromatin remodeling.

As mentioned above, when HPFH is co-inherited with β-hemoglobinopathies, it leads to a milder disease phenotype [120,199,200]. This observation has inspired therapeutic strategies aimed at reactivating γ-globin expression in adult erythroid cells (Figure 4). Such reactivation can compensate for defective or absent β-globin in β-thalassemia and inhibit β-globin polymerization in sickle cell disease (SCD), providing a versatile treatment strategy [201,202].

Multiple preclinical studies have used genome editing tools, including TALENs, CRISPR-Cas9, and base editors, to reactivate the developmentally silenced γ-globin [203] (Figure 4). One set of targets includes the intergenic region between the Aγ- and δ-globin genes, which contains a pseudogene (*HBBP1*) and a non-coding gene (*BGLT3*). Deletion of *HBBP1* reactivates γ-globin in adult-type human erythroid cells, suggesting a developmental silencing role [204]. *BGLT3* transcribes a long non-coding RNA that enhances chromatin looping between the LCR and the γ-globin genes, positively regulating their expression [205]. Several HPFH-associated mutations have been identified within the *HBBP1/BGLT3* locus [206,207]. 

In addition to these distal elements, more proximal regulatory regions within the γ-globin gene promoters have also been targeted to reactivate fetal hemoglobin. Another successful strategy involves direct editing of the γ-globin *(HBG)* promoters. TALEN-mediated disruption of regulatory elements in these promoters reactivates γ-globin expression ex vivo and in vivo in humanized mouse models [208]. Similarly, introduction of naturally occurring HPFH mutations—such as the Sicilian HPFH variant—using CRISPR-Cas9 leads to a twofold increase in γ-globin mRNA in HSPC-derived erythroid colonies [209]. 

These promoter-based strategies frequently act by disrupting the binding of BCL11A, a major transcriptional repressor of γ-globin. Notably, the BCL11A binding site overlaps with the region where naturally occurring HPFH mutations such as −115C > T, −114C > T, and −113A > G have been identified in the *HBG1* and *HBG2* promoters. These point mutations disrupt BCL11A binding, leading to elevated γ-globin expression in individuals with HPFH. Disruption of the BCL11A binding site at the γ-globin promoters via in vitro [210] or in vivo [211] CRISPR-Cas9 editing was shown to reactivate γ-globin expression. In addition to BCL11A, LRF (ZBTB7A) also contributes to γ-globin repression by binding directly to the promoters. Genome editing strategies that delete or mutate the LRF binding motif in the HBG promoters can relieve this repression, further enhancing fetal hemoglobin induction [125].

In addition to disrupting transcription factor binding at the γ-globin promoters, an alternative strategy targets a regulatory element upstream of BCL11A itself. *BCL11A* expression is controlled by multiple enhancers, one of which, located in intron 2 and consisting of three DHSs (+56, +58, +62), is selectively active in the erythroid lineage. Targeted disruption of a *GATA1* binding site [212,213] at the +58 site of this erythroid enhancer of *BCL11A* leads to reduced BCL11A expression in erythroid cells without affecting its function in other hematopoietic or immune lineages. This selective silencing enables robust reactivation of γ-globin while preserving HSC function, making it particularly attractive for therapeutic genome editing. Importantly, this strategy has demonstrated efficacy in human HSPCs ex vivo and in animal models [213,214,215,216] and has advanced not only to clinical trials [217,218,219] but also to regulatory approval and commercialization [220]. There are two phase 1/2/3 clinical trials funded also by VERTEX-Therapeutics in collaboration with CRISPR-Therapeutics for TDT beta-thalassemia (CLIMB THAL-111, NCT03655678) and sickle cell disease patients (CLIMB SCD-121, NCT03745287). In these trials, the disruption of the erythroid enhancer of BCL11A using the CRISPR-Cas9 system resulted in sustained normal Hb levels 12.5±1.8 g/dl for SCD and 13.1±1.4 g/dl for beta- thalassemia patients After the gene therapy, the 91% of the thalassemic patients were transfusion independent and the 97% of the SCD had reduced vaso-occlusive crisis for a period of 12 months or more [217,218,219].

Building on CRISPR-Cas9 technology, recent advances have led to the development of base editors, which allow the introduction of precise point mutations without creating double-strand breaks [221,222,223]. Using a cytidine base editor (CBE), HPFH-associated mutations at −115 C or −114 C in the *HBG* promoters were successfully introduced, leading to increased γ-globin expression in HSPCs from both healthy donors and patients with β-thalassemia [224]. Similarly, adenine base editors (ABEs) have been used to introduce the −113 A > G substitution at *HBG* promoters, resulting in elevated γ-globin expression in β-YAC/CD46tg mice and in HSPCs derived from β-thalassemia and SCD patients [225,226]. Notably, CBEs and ABEs have also been applied to disrupt LRF binding sites or to generate de novo *KLF1* binding sites, promoting γ-globin reactivation and preventing sickling both during ex vivo erythroid differentiation and in vivo in humanized SCD mouse models [227]. More recently, Myuranathan et al. used an ABE to introduce a −175 A > G mutation that creates a *GATA*-*TAL1* motif at the *HBG* promoters. This edit did not compromise the engraftment potential of modified cells and resulted in effective HbF reactivation both ex vivo and in vivo [228]. 

Beyond DNA editing, epigenome editing technologies have enabled the modulation of gene expression without altering the underlying DNA sequence. A notable example involves targeting the *HBG* promoters with catalytically inactive Cas9 (dCas9) proteins fused to the p300 histone acetyltransferase domain. The dCas9-p300 complexes, derived from various Cas9 orthologs, successfully increased *HBG* expression in 293FT cells, highlighting the potential of epigenetic reprogramming in hemoglobinopathies [229].

### 5.3. Epigenetic Therapeutics in Erythroid Disorders 

Epigenetic modifiers represent a versatile class of therapeutic agents with the potential to modulate gene expression without altering the underlying DNA sequence. Classes of such agents, including DNA Methyltransferase Inhibitors (DNMT inhibitors), Histone Deacetylase Inhibitors (HDAC inhibitors), Histone Methyltransferase (HMT) or Demethylase Inhibitors, and Bromodomain and Extra-Terminal Domain (BET) Inhibitors, have been evaluated in various erythroid disorders [230]. A major therapeutic goal has been the reactivation of fetal hemoglobin (HbF) in β-hemoglobinopathies such as sickle cell disease and β-thalassemia. Pharmacologic reactivation of γ-globin expression offers a non-invasive strategy to compensate for defective or absent β-globin chains (Figure 4). DNMT inhibitors such as azacytidine, decitabine, and the DNMT1-selective inhibitor GSK3482364 have demonstrated therapeutic potential in β-hemoglobinopathies by inducing γ-globin expression through promoter hypomethylation, as shown in patients with β-thalassemia and SCD as well as in transgenic SCD mouse models [231,232,233,234]. Moreover, HDAC inhibitors such as butyrate and trichostatin A led to gamma-globin reactivation, through affecting p38 MAPK and STAT-5 signaling pathway [231,232]. The ACY-957, HDAC1/2 inhibitor efficiently reactivates HbF expression in SCD patients’ cells by histone acetylation-induced activation of the *GATA2* gene [235]. UNC0638, a Histone Methyltransferase (HMT) Inhibitor, decreases the accumulation of H3K9me2 upstream of gamma-globin, enhancing the loop formation between gamma-globin promoters and LCR locus and promoting gamma-globin reactivation [236,237,238,239] (Figure 4). Notably, the RK-701 inhibitor of the euchromatic histone/lysine N-methyltransferase 2 (EHMT2) reactivates gamma-globin expression by increasing *BGLT3* expression [240]. Moreover, lysine-specific demethylase 1 (LSD1) inhibitors, such as tranylcypromine (TCP), RN-1, and ORY-3001 have been widely employed for the pharmacological induction of gamma-globin expression [241]. TCP and RN-1 increase H3K4 di- and tri-methyl lysine at the γ-globin promoter and efficiently reactivating gamma-globin expression in SCD mouse and baboon models [242], while oral administration of ORY-3001 further enhances the effect in vivo [243]. Additionally, peroxisome proliferator–activated receptor γ coactivator-1α, (PGC-1α) triggers gamma-globin expression through its interaction with nuclear receptors testicular receptor 2/4 (TR2/TR4). PGC-1α activation by SR-18292 or the ZLN005 agonist, led to increased percentage of F-cells in normal CD34+ cells, β-YAC transgenic, and SCD mice, reducing splenomegaly and sickling, especially when used in combination with hydroxyurea [244,245]. 

Beyond β-hemoglobinopathies, epigenetic dysregulation also plays a central role in erythroid malignancies, such as acute erythroid leukemia (AEL). Acute erythroid leukemia is an uncommon and aggressive subtype of AML, representing <1% of adult AML cases. It is typified by predominant erythroblast proliferation, frequent biallelic *TP53* mutations, complex cytogenetics, and poor prognosis [145,146,147,148,149,246,247]. Large-scale molecular studies have uncovered recurrent alterations across epigenetic regulators—including *DNMT3A*, *TET2*, *NSD1*, *BCOR*—and splicing factors, which synergize with *TP53* loss to impair erythroid differentiation via widespread DNA methylation and histone modification changes affecting loci such as *GATA1*, *TAL1*, *KLF1*, and *EPOR* [247]. Mouse models and functional analyses confirm that combined lesions (e.g., *TP53* + *BCOR* + *DNMT3A*) induce erythroid-skewed acute leukemia, driven by genome-wide hypomethylation at erythroid regulatory elements; such preclinical studies also highlight potential sensitivities to CDK7/9 inhibitors, PARP inhibitors, and menin-bromodomain targeting agents [145,149,246,247] (Table 1).

## 6. Conclusions and Future Directions

Erythropoiesis is governed by complex, multilayered regulatory mechanisms involving dynamic chromatin remodeling, lineage-defining transcription factors, and stage-specific regulatory elements. This review has outlined how these epigenetic processes coordinate erythroid lineage commitment, differentiation, and globin gene expression and how their disruption contributes to a spectrum of hematologic diseases, from β-hemoglobinopathies to erythroid malignancies.

Recent advances in genomics, gene editing, and epigenetic profiling have not only deepened our understanding of erythroid biology but have also enabled the development of precision therapies, ranging from fetal hemoglobin reactivation to enhancer-driven gene therapy. These therapeutic strategies increasingly reflect the regulatory logic of the erythroid program itself, offering promising avenues for targeted and durable interventions.

While this review focuses on key aspects of epigenetic regulation in steady-state erythropoiesis, it is important to acknowledge that this represents only a fraction of the regulatory complexity underlying red blood cell development. Important areas such as the contribution of non-coding RNAs, post-translational modifications of epigenetic regulators, and the unique regulatory dynamics of stress erythropoiesis remain beyond the scope of this article. Continued integration of these dimensions will be essential to build a more complete and nuanced understanding of erythroid biology in both health and disease.

## Figures and Tables

**Table 1:** Summary of epigenetic agents, their mechanisms, and effects in erythroid disorders.

Epigenetic Agent Class	Example Compounds	Mechanism or Target	Therapeutic Effect
DNMT Inhibitors	Azacytidine, Decitabine, GSK3482364	Hypomethylation of γ-globin promoters; DNMT1 selective inhibition	Reactivation of γ-globin in β-thalassemia and SCD
HDAC Inhibitors	Butyrate, Trichostatin A, ACY-957	Affect p38 MAPK and STAT5 signaling; histone acetylation; *GATA2* activation	Reactivation of γ-globin in SCD
HMT Inhibitors	UNC0638, RK-701	Decrease H3K9me2; promote chromatin looping; increase *BGLT3* expression	Enhanced γ-globin reactivation
BET Inhibitors	Apabetalone (RVX-208), JQ1, I-BET762 (Molibresib)	Target bromodomain and extra-terminal domains	Increased γ-globin expression and HbF levels in SCD and β-thalassemia models
LSD1 Inhibitors	TCP, RN-1, ORY-3001	Target LSD1	Reactivation of γ-globin in SCD, β-YAC mice and baboons
PGC-1a modulators	SR-18292, ZLN005	Increase PGC1a expression	Increased γ-globin expression in human primary cells, SCD, and β-thalassemia mouse models

Abbreviations: DNMT: DNA methyltransferase, HDAC: Histone deacetylase, HMT: Histone methyltransferase, BET: Bromodomain and extra-terminal domain, LSD1: Lysine-specific demethylase 1, PGC-1α: Peroxisome proliferator-activated receptor gamma coactivator 1-alpha, HbF: Fetal hemoglobin, SCD: Sickle cell disease, β-YAC: Beta yeast artificial chromosome, p38 MAPK: p38 mitogen-activated protein kinase, STAT5: Signal transducer and activator of transcription 5, H3K9me2: Histone H3 lysine 9 dimethylation.

## Data Availability

No data were generated for this article.
